# Bcl-2 and Bcl-xL in Diabetes: Contributions to Endocrine Pancreas Viability and Function

**DOI:** 10.3390/biomedicines13010223

**Published:** 2025-01-17

**Authors:** Atenea A. Perez-Serna, Daniel Guzman-Llorens, Reinaldo S. Dos Santos, Laura Marroqui

**Affiliations:** 1 Instituto de Investigación, Desarrollo e Innovación en Biotecnología Sanitaria de Elche (IDiBE), Universidad Miguel Hernández de Elche, 03202 Elche, Alicante, Spain; atenea.perezs@umh.es (A.A.P.-S.); dguzman@umh.es (D.G.-L.); 2 CIBER de Diabetes y Enfermedades Metabólicas Asociadas, Instituto de Salud Carlos III, Madrid, Spain; 3 Unidad de Investigación, Fundación para el Fomento de la Investigación Sanitaria y Biomédica de la Comunidad Valenciana (FISABIO), Hospital General Universitario de Elche, Camí de l’Almazara 11, 03203 Elche, Alicante, Spain

**Keywords:** apoptosis, Bcl-xL, Bcl-2, diabetes, hormone secretion, pancreatic β-cells

## Abstract

Diabetes is a chronic metabolic disorder whose prevalence increases every year, affecting more than 530 million adults worldwide. Type 1 (T1D) and type 2 diabetes (T2D), the most common forms of diabetes, are characterized by the loss of functional pancreatic β-cells, mostly due to apoptosis. B-cell leukemia/lymphoma 2 (Bcl-2) and B-cell lymphoma-extra large (Bcl-xL), two anti-apoptotic proteins belonging to the Bcl-2 family, are crucial for regulating the intrinsic pathway of apoptosis. However, over the years, they have been implicated in many other cellular processes, including intracellular Ca^2+^ homeostasis and the regulation of mitochondrial metabolism. Thus, understanding the biological processes in which these proteins are involved may be crucial to designing new therapeutic targets. This review summarizes the roles of Bcl-2 and Bcl-xL in apoptosis and metabolic homeostasis. It focuses on how the dysregulation of Bcl-2 and Bcl-xL affects pancreatic β-cell function and survival, and the consequences for diabetes development.

## 1. Introduction

Diabetes is a chronic metabolic disorder characterized by elevated blood glucose levels, also known as hyperglycemia, which stems from a deficiency in insulin secretion, insulin action, or both. According to the 2021 International Diabetes Federation report, diabetes currently affects 537 million adults worldwide, and it has been projected that approximately 783 million will be living with diabetes by 2045 [1].

Type 1 (T1D) and type 2 diabetes (T2D), the most common forms of diabetes, are characterized by the loss of functional pancreatic β-cells, which are responsible for the synthesis and release of insulin, the main hormone involved in the regulation of blood glucose levels. Regarding their pathogenesis, T1D is the result of an autoimmune assault that culminates in islet inflammation and β-cell death, whereas T2D derives from mild-to-moderate β-cell loss due to metabolic stress (e.g., glucolipotoxicity) [2,3,4]. Despite their fundamentally different etiologies, a growing body of data suggests that T1D and T2D share common features concerning their development and progression. The onset of both diseases seems to mainly arise from a complex combination of a predisposing genetic background and environmental triggers [5,6]. While in T1D viral and bacterial infections, as well as dietarian immunogens, have been reported to induce or potentiate autoimmunity [7], T2D is primarily promoted by an obesogenic diet and lifestyle changes that can be worsened by exposure to environmental pollutants [8,9].

In T1D and T2D, pancreatic β-cell mass dysfunction and apoptosis are the aftermaths of stress responses to several insults, such as viral infections, proinflammatory cytokines, and free fatty acids [3]. These triggers activate stress-responsive pathways, including endoplasmic reticulum (ER) stress, oxidative stress response, autophagy, and cellular senescence, which are regulated by cell-specific and context-dependent transcription factors and gene/protein networks [10]. For example, the transcription factor nuclear factor-κB (NF-κB) is pro-apoptotic in β-cells [11,12] but has protective effects in other cell types [13].

The B-cell leukemia/lymphoma 2 (Bcl-2) family of proteins is crucial for the survival of pancreatic β-cells. The balance between pro- and anti-apoptotic members of the family in response to stress is a tightly regulated and dynamic process that determines whether cells undergo apoptosis via the intrinsic mitochondrial pathway or survive [14]. The crucial role of the Bcl-2 family of proteins in the cell fate has made them interesting actors in the search for pro-death therapies in cancer [15,16], and pro-survival therapies in diseases affecting cell types with low proliferation rates, such as neuronal pathologies [17,18] and diabetes [19]. The purpose of this review is to analyze the cell-specific and context-dependent roles of two anti-apoptotic members of the Bcl-2 family of proteins, namely, Bcl-2 and B-cell lymphoma-extra large (Bcl-xL). Moreover, we aim to shed light on recent research regarding their roles in β-cells. Finally, we discuss some limitations, gaps in the current knowledge, and potential therapeutic opportunities related to these pro-survival proteins in the context of β-cells and diabetes.

## 2. Function and Structure of Bcl-2 and Bcl-xL

### 2.1. Homology of Bcl-2 Proteins

The proteins belonging to the Bcl-2 family are crucial regulators of the cell cycle and survival. The anti-apoptotic protein Bcl-2 was the first member to be described in cancerous lymphocytic B-cells, where its exacerbated steady expression was related to increased cell viability and acute lymphocytic B-cell leukemia [20].

Since its discovery, 25 homologues of Bcl-2 have been described [21]. These homologues are divided into three categories, depending on the Bcl-2 homology (BH) domains present in their structure and their function: (1) anti-apoptotic proteins (Bcl-2, Bcl-xL, Bcl-W, Bcl-B, Bfl-1, and Mcl-1L), all harboring the four highly conserved BH regions (BH1, BH2, BH3, and BH4); (2) pro-apoptotic proteins (Bax, Bak, Bok, and Bcl-xS), which also share the four conserved BH1-4 domains; and (3) pro-apoptotic proteins exclusively presenting a single BH3 domain, also known as BH3-only proteins. This last category can be subdivided into BH3-only sensitizers (Bad, Bik, Bmf, Bnip3, Hrk/DP5, Beclin-1, and Noxa) and BH3-only activators (Bim, tBid, Mule, and Puma). Of note, several anti-apoptotic members of the family lack the BH4 domain, such as Mcl-1, Bfl-1/A1, and Bcl2L121 [21,22]. Additionally, other BH-3-only isoforms, namely, Bcl-Rambo, Bcl-G, Mcl-1S, and Mcl-1ES, have been proposed as potentially pro-apoptotic [23,24,25].

The *BCL2* gene, located on chromosome 18, renders three mRNA variants that translate into two splicing alternatives: Bcl-2α, which consists of 239 amino acids and encodes the active, membrane-bound isoform; and Bcl-2β, which has only 205 residues due to a truncation of the hydrophobic tail at the C-terminus and lack the transmembrane domain [26,27]. Although the anti-apoptotic activity of the Bcl-2β isoform is still controversial [21], recent evidence suggests that it might be involved in promoting tumor angiogenesis [28].

Bcl-xL is one of the two isoforms yielded by alternative splicing of exon 2 of the *BCL2L1* gene (Bcl-2 like 1), located on chromosome 20q11.21. The long, anti-apoptotic isoform Bcl-xL, which contains 233 amino acids, results from the selection of the proximal 5′ site on exon 2. In contrast, the short, pro-apoptotic isoform Bcl-xS is translated from the distal 5′ splicing site and contains 170 amino acids [29]. Due to its role as an antagonist of the anti-apoptotic function of Bcl-xL and Bcl-2, the alternative splicing of Bcl-xS is tightly regulated to favor a high Bcl-xL/Bcl-xS ratio under normal conditions [30].

### 2.2. Structure of Bcl-2 and Bcl-xL

Curiously, the crystallographic and solution structures of Bcl-xL [31] were established before those of Bcl-2 [32]. Briefly, Bcl-xL consists of two central hydrophobic α-helices, surrounded by amphipathic helices. Helices α1 and α2 are connected to a flexible loop of 60 residues, which is not essential for Bcl-xL anti-apoptotic activity. The BH1, BH2, and BH3 regions are spatially close, forming an elongated hydrophobic groove that may act as the binding site for interacting members of the Bcl-2 family [31].

Regarding the two Bcl-2 isoforms, their structures consist of six α-helices with a surface hydrophobic pocket similarly to Bcl-xL. Comparisons between Bcl-2 and Bcl-xL structures show that, although the overall fold is the same, their structural topology and electrostatic potential of the binding groove present some differences [32]. In Bcl-2α, the 22 amino acids in the COOH-terminal act as a signal-anchor sequence responsible for its integration into the outer mitochondrial membrane [33], but it is not essential for its anti-apoptotic effects [34].

### 2.3. Preferential Localization of Bcl-2 and Bcl-xL

In the early 1990s, a series of studies established that Bcl-2 was localized to the inner mitochondrial membrane, more specifically, at the interacting points between the external and internal mitochondrial membranes [33,35,36]. Later research, however, indicated that it is situated on the outer mitochondrial membrane [37,38,39,40,41]. Additionally, subsequent studies revealed the presence of Bcl-2 not only in the mitochondria, but also in the nuclear outer membrane, the nucleus, and the ER [42]. Similarly to Bcl-2, Bcl-xL is primarily localized to the outer mitochondrial membrane [14,43] but can also be found in the inner mitochondrial membrane, in the cytosol, or bound to the ER [44,45]. Many of these studies were performed in B-cell lymphoma cell lines [35,37,38,39,40,41,43,45]. However, this localization seems to be conserved among cell types, including heart [33], liver [36] and pancreatic β-cells [46,47,48]. Specifically, Luciani et al. described a strong correlation between Bcl-xL and mitochondrial membranes in β-cells, which was higher than its ER localization and Bcl-2 mitochondrial localization [47] in MIN6 cells. Later, Aharoni and colleagues confirmed Bcl-2 localization in the inner mitochondrial membrane of MIN6 cells and primary β-cells from C57/BL6 mice [46], in line with human islet reports that indicated a microsomal localization of the Bcl-2 anti-apoptotic protein [48].

The anti-apoptotic role of Bcl-2 might exhibit spatial dependence, varying according to the specific apoptotic pathway involved. For instance, ER-bound Bcl-2 is particularly effective in inhibiting starvation/myc-induced apoptosis, where a loss in mitochondrial membrane potential precedes the release of cytochrome *c* from mitochondria. In contrast, only Bcl-2 localized to the outer mitochondrial membrane can counteract etoposide-induced apoptosis, characterized by an early release of cytochrome *c* before the loss of mitochondrial membrane potential [49].

### 2.4. Interactions Within the Family of Bcl-2 Proteins

The complex interactions between pro- and anti-apoptotic members of the Bcl-2 family play a pivotal role in determining whether cells survive or undergo cell death through mitochondrial-mediated apoptosis, also known as the intrinsic pathway of apoptosis, in response to pathophysiological challenges. Classically, the role played by Bcl-2 and Bcl-xL in the regulation of the intrinsic pathway of apoptosis is known as their canonical function. As this canonical role has been previously reviewed elsewhere [50,51,52], here we will focus on how these proteins interact with other members of the Bcl-2 family involved in the intrinsic pathway of apoptosis.

**Figure 1:**
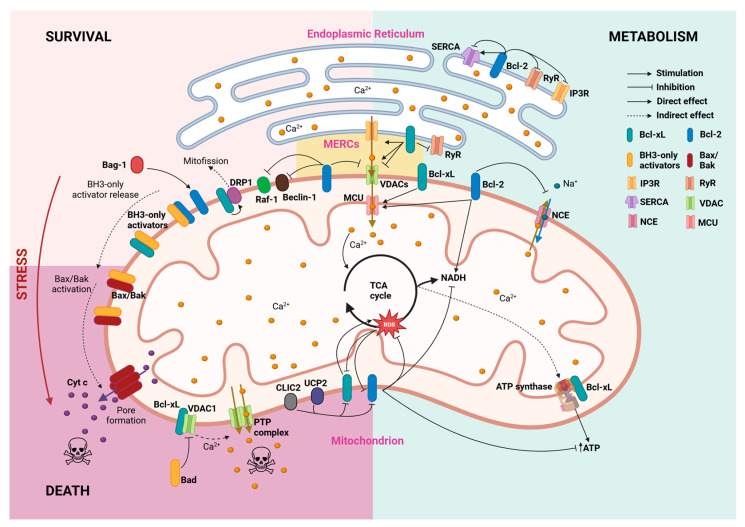
Survival and metabolic homeostasis maintenance by Bcl-2 and Bcl-xL proteins. Through multiple interactions with mitochondrial and ER proteins, Bcl-2 (blue) and Bcl-xL (green) establish a network of processes that connect survival promotion with Ca^2+^ homeostasis and fluxes between both organelles. Arrows depict direct interactions, while dashed arrows refer to indirect effects reported in the literature. MERCs: mitochondria-endoplasmic reticulum contact sites; MCU: mitochondrial calcium uniporter; NCE: Na+-Ca2+ exchanger; PTP: permeability transition pore.

Under basal conditions, BH3-only activators are sequestered in a heterodimer structure by pro-survival members of the Bcl-2 family, such as Bcl-2 and Bcl-xL. This inhibition occurs by direct interaction between the BH3 domain of BH3-only sensitizers and the highly conserved BH3 domain-binding pocket in both pro- and anti-apoptotic members of the family [53]. In the presence of apoptotic signals, these BH3-only activators are released from the anti-apoptotic proteins upon displacement by BH3-only sensitizers and are free to bind to pro-apoptotic partners of the family (Figure 1).

The interactions between Bcl-2 family proteins are determined by their abundance in each locus of action and the affinity between partner proteins [14]. Different models describe this process, among which the “embedded together” model emphasizes the importance of the intracellular membrane where these proteins are attached [54].

The interaction with activated BH3-only proteins induces a conformational change in the apoptotic effectors Bax/Bak, that disfavors their binding to anti-apoptotic Bcl-2 proteins, and which results in their homo-oligomerization and pore-formation in the outer membrane of the mitochondria.

Despite their high homology, Bcl-xL and Bcl-2 present different interactions with their associated pro-apoptotic antagonists, such as Bcl-2-associated death promoter (Bad) and Bcl-2-associated X protein (Bax) [55]. Computational interaction entropy analyses performed by Duan and colleagues found a higher affinity between Bcl-xL and Bad than Bcl-xL and Bax, which was also the case for Bcl-2. These researchers also identified the key residues in Bcl-xL complexes (arginine 104, tyrosine 105, leucine 116, and leucine 134) and Bcl-2 complexes (arginine 107, tyrosine 108, phenylalanine 112, glutamine 118, leucine 137, arginine 146, and tyrosine 202) with their pro-apoptotic counterparts [55].

In spite of the fact that the regulation of anti- and pro-apoptotic members of the Bcl-2 family occurs through direct interaction between their BH3 domains [56], the transmembrane domain of Bcl-2 proteins can mediate these interactions. In this line, Beigl and collaborators recently found that Bcl-2 regulates Bok-induced apoptosis through the direct interaction of their C-terminal transmembrane domains at the ER membrane. In fact, the Bcl-2 inhibition of Bok specifically depends on this interaction [57]. Furthermore, the functionality of the Bcl-xL transmembrane domain is also a novel matter of research. A recent study by Wu and colleagues found that Bcl-xL transmembrane domain shows strong anti-apoptotic activity on its own, comparable to the full-length protein, being able to directly inhibit Bad and Bax at the mitochondrial membrane of living HeLa cells [58].

Furthermore, the phosphorylation status seems to have a differential role in Bcl-xL and Bcl-2 proteins. While the phosphorylation of the serine residue at position 70 is crucial for the Bcl-2 full pro-survival phenotype [59], multiple-site phosphorylation can have a pro-apoptotic effect. Serum-starved cells presenting multiple Bcl-2 phosphorylations—including serine 70 among others—lose the Bcl-2 autophagy inhibition as phosphorylated Bcl-2 is unable to interact with the major autophagy regulator Beclin-1 [60]. On the other hand, the phosphorylation of the Bcl-xL serine-14 residue has an inhibitory effect, favoring its detachment from Bax [61]. Moreover, the phosphorylation of the serine-62 residue in Bcl-xL has been shown to reduce its ability to bind and retain Bax [62], instead causing it to interact with the cyclin-dependent kinase 1 to arrest the cell cycle during the G_2_ checkpoint [63].

### 2.5. Non-Canonical Functions of Bcl-2 and Bcl-xL

Besides their canonical roles in the hierarchical regulation of mitochondrion-dependent apoptosis, Bcl-2 proteins are also involved in other cellular processes, mainly through the regulation of Ca^2+^ homeostasis [64,65]. In this review, we will refer to these apoptosis-independent functions as the “non-canonical” roles of Bcl-2 and Bcl-xL.

#### 2.5.1. ER Ca^2+^ Homeostasis

Many studies report that Bcl-2 and Bcl-xL can control intracellular Ca^2+^ homeostasis through different and complex pathways [66,67]. Both proteins are present at the surface of the two main cell Ca^2+^ reservoirs: the ER and the mitochondria. Moreover, as will be further discussed in this review, their interaction with both ER and mitochondrial Ca^2+^ machinery, together with their localization at the mitochondrial-associated ER membranes [68], suggests that these proteins could participate in the modulation of the ER–mitochondrial Ca^2+^ trafficking (Figure 1). In this line, a study from Williams and colleagues showed that Bcl-xL interacts with 1,4,5-trisphosphate receptor 3 (IP3R) type 3 at the mitochondrial-associated ER membrane to enhance transient ER-to-mitochondrial Ca^2+^ trafficking, dynamically modulating cell metabolism in CHO cells [69].

In contrast, ER-localized IP3R regulation by both Bcl-2 and Bcl-xL has been extensively studied [70,71,72]. Bcl-2 interaction with IP3R promotes survival by diminishing ER Ca^2+^ trafficking towards the cytosol and mitochondria through a BH4-domain dependent mechanism [70,71,72]. This effect has been narrowed to the Bcl-2α isoform [73]. Even though the ER Ca^2+^ lowering effects of Bcl-2 might seem contradictory to its anti-apoptotic nature, several hypotheses have arisen to explain this effect. One hypothesis suggests that modest Ca^2+^ intraluminal concentrations lead to reduced ER stress-induced Ca^2+^ mobilization and decreased mitochondrial Ca^2+^ uptake [74,75]. Supporting this hypothesis, Pinton and colleagues found that Bcl-2-mediated ER Ca^2+^ depletion prevented the ceramide-induced increase in cytoplasmic Ca^2+^ and mitochondrial damage, which protected HeLa cells from apoptosis [76]. On the other hand, the Bcl-2-induced reduction of Ca^2+^ release might not be related to a decrease in the ER Ca^2+^ pool but to the inhibition of IP3R and, therefore, reduction in Ca^2+^ efflux. In 1997, He and colleagues demonstrated that Bcl-2 prevented the depletion of the ER Ca^2+^ pool in mouse lymphoma cells [77]. Despite their differences, both hypotheses emphasize that the anti-apoptotic effect of Bcl-2 resides in its control of the ER-induced cytosolic Ca^2+^ oscillations.

The Bcl-xL interaction with IP3R has shown both functional and survival outcomes [70,78,79,80]. On the one hand, Bcl-xL interacts with the C-terminus of IP3R, sensitizing the channel to lower IP3 concentrations and thus enhancing both Ca^2+^- and IP3-dependent regulation of the channel. This effect is accompanied by a reduction in the ER Ca^2+^ content, the consequent sensitization of ER Ca^2+^ oscillations to extracellular signals, and increased mitochondrial bioenergetics, suggesting that Bcl-xL mediates ER–mitochondrial Ca^2+^ flux [79]. Recently, a study by Nakamura and colleagues deepened this issue and demonstrated that the ability of Bcl-xL to modulate the IP3R function depends on its phosphorylation status [61]. Alternatively, the Bcl-xL inhibitory effect on IP3R activity has a protective effect against Ca^2+^-driven apoptosis [81]. Furthermore, the inhibitory effect of Bcl-xL on IP3R extends to the regulation of the receptor expression [82]. An analysis of Bcl-xL effects on each of the three isoforms of IP3R, performed by Li and colleagues, demonstrated that while Bcl-xL only reduced ER Ca^2+^ storage in IP3R3-expressing cells, its enhancement of Ca^2+^ signaling was isoform-independent. Therefore, the pro-survival effects of Bcl-xL on IP3R are conferred through increasing the spontaneous intracellular Ca^2+^ oscillations rather than ER Ca^2+^ content dampening [83].

Ryanodine receptors (RyRs) are the other co-protagonists of receptor-mediated ER Ca^2+^ dynamics [84]. As with IP3R binding, the BH4 domain of Bcl-2 is essential for its interaction with RyRs [85]. In contrast to Bcl-2, Bcl-xL requires the combined contribution of its BH4 and BH3 domains to fully bind to RyR channels [86]. Importantly, regardless of the specific binding mechanism, Bcl-2 and Bcl-xL binding to RyRs leads to a decrease in RyR activity, thereby inhibiting RyR-mediated ER Ca^2+^ release [85,86] (Figure 1).

The Bcl-2 effect on the Ca^2+^ levels inside the lumen of the ER has also been hypothesized to act through the Bcl-2 regulation of store-operated Ca^2+^ entry [87] or through direct effects of Bcl-2 on the sarcoplasmic/endoplasmic reticulum Ca^2+^-ATPase (SERCA). The ability of Bcl-2 to control ER Ca^2+^ is directly related with its cell survival function, as phosphorylated forms of Bcl-2 are unable to lower ER Ca^2+^ content or interact with BH3-only members of the family [88]. To our knowledge, no relationship between Bcl-xL and SERCA has been reported. However, the relationship between Bcl-2 and the sarcoplasmic Ca^2+^ pump has been a matter of study since the late 1990s. In 1998, Kuo and colleagues reported that clonal Bcl-2 expression increased SERCA2 expression, promoting increased ER Ca^2+^ uptake [89]. Nonetheless, a truncated form of Bcl-2 directly inhibits SERCA activity in rat skeletal muscle. This inhibition was associated with a partial unfolding of SERCA, yet it did not involve proteolytic degradation or an increased sensitivity of SERCA to oxidation [90]. Together, these results show that differing functions of ER-related Bcl-2 may occur depending on cell context.

#### 2.5.2. Mitochondrial Homeostasis

Along with the ER Ca^2+^ storages, the mitochondrial Ca^2+^ content is crucial for regulating cell survival and maintaining the Ca^2+^ reservoir [91,92]. The Ca^2+^ flux across the outer mitochondrial membrane occurs mainly through the voltage-dependent anion channels (VDACs), whose isoforms can transfer Ca^2+^ with different functional implications [93]. Bcl-xL was the first Bcl-2 protein to be described as able to interfere with VDACs by Craig Thompson and colleagues. These researchers provided evidence that Bcl-xL sustained coupled respiration and ATP production in growth factor-deprived cells by maintaining VDACs in an open state [94,95]. When the BH3-only protein Bad is dephosphorylated, it displaces Bcl-xL from VDAC1. Once liberated, VDAC1 forms the mitochondrial permeability transition pore, leading to the depletion of mitochondrial Ca^2+^ [96,97]. Furthermore, it has been stated that Bcl-xL direct interaction with VDCA1 and the less studied VDCA3 promotes mitochondrial Ca^2+^ intake [98]. In contrast, the BH4 domain from Bcl-xL, but not from Bcl-2, limits VDAC1 Ca^2+^ permeability, thus restricting the Ca^2+^ uptake by the organelle and inhibiting cell death [99,100]. For its part, Bcl-2 interaction with the N-terminal α-helix of VDAC1 inhibits channel function and has a cytoprotective effect [101].

The disparity of effects of Bcl-2 and Bcl-xL on mitochondrial Ca^2+^ status has been hypothesized to be a consequence of their implications in cell survival. In this model, under physiological conditions, Bcl-xL and Bcl-2 would promote mitochondrial Ca^2+^ uptake to stimulate bioenergetics, whereas under pro-apoptotic stimuli, they might inhibit the VDAC to prevent lethal Ca^2+^ overload in the mitochondria [93]. Following this hypothesis, it would be argued that Bcl-2 anti-apoptotic members could interfere in other mitochondrial Ca^2+^ exchange pathways. This extension of the model is supported by the results of Zhu and colleagues, who demonstrated that Bcl-2 overexpression increases mitochondrial Ca^2+^ matrix content by reducing the mitochondrial Na^+^-Ca^2+^ exchange in cardiomyocytes [102] (Figure 1).

Another crucial process in mitochondria homeostasis is the regulation of mitochondrial dynamics (i.e., mitochondrial fusion and fission) and mitophagy, of special relevance for neuronal physiology [103].

The protein dynamin-related protein 1 (Drp1) plays a crucial part in mitochondrial fission. In hippocampal neurons, Bcl-xL ensures neuronal survival and function by increasing the synaptic localization of mitochondria and acts through Drp1 to promote mitochondrial fission and the consequent mitophagy of damaged mitochondria [104]. Similarly to its role in the regulation of VDAC activity, Bcl-xL shows a dual effect on mitochondrial fission, as its counteraction of Bax and Bak inhibits mitochondrial-like membrane pore formation, budding, and fission in in vitro models [105]. Even though the Bcl-xL inhibition of Bax and Bak abolishes cytochrome *c* release, it is unable to counteract Bax- and Bak-initiated mitochondrial network remodeling in HeLa cells [106]. These findings suggest that Bcl-xL effects on mitochondrial network dynamics are the results of multiple factors.

Along with the promotion of the open state of VDACs discussed above, Bcl-xL control over mitochondrial dynamics might indirectly promote reactive oxygen species (ROS) production [107] and ATP production, which has already been proposed to contribute to neural plasticity [108]. For instance, Alavian and colleagues found out that Bcl-xL directly interacts with the mitochondrial F_1_F_O_-ATP synthase β-subunit, increasing metabolic efficiency by reducing ion leak and thus improving net H^+^ transport [109]. These findings were later confirmed by Chen and colleagues [110]. Furthermore, a decreased expression of Bcl-xL in hippocampal neurons reduces mitochondrial motility and ATP retainment, enhancing their vulnerability to excitotoxic insults [111]. Finally, and consistent with the previously mentioned results, the pro-metastatic effects of Bcl-xL and Bcl-2 in breast cancer cells can be at least partially attributed to their pathological promotion of VDAC activity, which is accompanied by mitochondrial Ca^2+^ uniporter increased activity and consequent ATP production [112].

Recent research has shown that the Bcl-xL regulatory effect on ATP synthase is shared with the anti-apoptotic Bcl-2 family member Mcl-1 but not with Bcl-2 itself [113]. This seems contradictory considering Bcl-2 ability to arrest both ATP and ROS production to delay cell cycle progression to S phase entry [114] (Figure 1). Later proteomics research by the same authors showed that the Bcl-2 arrest of the cell cycle progression from G0/G1 to S phase entry occurs via the downregulation of ribosomal dynamics and oxidative phosphorylation. These authors found that in serum-deprived fibroblasts, Bcl-2 decreased the expression of transcripts related to mitochondrial Ca^2+^ homeostasis, while the electron transport chain and response to nutrients pathways were upregulated [115]. The authors observed changes in NADH dehydrogenase, cytochrome *c* reductase/oxidase, and mitochondrial ATPase expression in Bcl-2-overexpressing fibroblasts. Curiously, in the case of ATPase, Bcl-2 expression upregulated the F subunit, while downregulating the B, H, and D subunits. These results indicate that Bcl-2 takes control of oxidative phosphorylation pathways, regulating ATP synthesis to counteract energy deficiency during the cell cycle arrest. Interestingly, while Bcl-2 proteins can regulate ROS production, that is a two-way street in which ROS increases have been shown to reduce the expression of Bcl-2, and both Bcl-2 and Bcl-xL phosphorylation in squamous cell carcinoma cells [116].

While Bcl-xL promotes the mitophagy of damaged mitochondria, Bcl-2 ensures that autophagy levels remain within the physiological range. It does so by inhibiting Beclin-1 [117]. Additionally, the Bcl-2 protein participates in a pathway that ensures mitochondrial integrity via Raf-1 sequestering to the outer mitochondrial membrane [118]. Interestingly, the modulation of Raf-1 expression via both Bcl-2 and Bcl-xL has a regulatory effect on cell differentiation, conducting hematopoietic differentiation towards myeloid or erythroid lineage [119].

In conclusion, Bcl-2 and Bcl-xL effects on mitochondrial homeostasis are not limited to the counteraction of pro-apoptotic signals. Contrarily, these members of the Bcl-2 family exert control on mitochondrial ATP production and Ca^2+^ homeostasis, ensuring a bioenergetic balance compatible with life.

## 3. Bcl-2 and Bcl-xL in Pancreatic Islets

Both in T1D and T2D, the clinical presentation of the disease is a consequence of the reduction in functional pancreatic β-cell mass below the threshold necessary for maintaining glucose homeostasis. As previously reviewed by Gurzov and Eizirik, the Bcl-2 family of proteins plays an important role in the β-cell demise in both T1D and T2D [52]. The pro-apoptotic members of this family Bax, Bad, Bak, DP5 and Puma have become classical markers of programmed cell death activation by proinflammatory cytokines and free fatty acids in β-cells [120,121,122,123]. In addition, the anti-apoptotic members of the family have also been studied in the context of β-cell survival. Lipotoxicity, a classical T2D insult, has been shown to downregulate Bcl-2, Bcl-xL, Mcl-1, and Bcl-w [124,125] in β-cells. Mirroring the establishment of Bax, Bad, Bak, DP5, and Puma as cell death standards, Mcl-1, Bcl-w, Bcl-2, and Bcl-xL have been used as cell survival markers in β-cell studies [126]. In this section, we will summarize what is currently known about the canonical (apoptosis-dependent) and non-canonical (apoptosis-independent) roles of Bcl-2 and Bcl-xL in different pancreatic cells.

### 3.1. Bcl-2 and Bcl-xL Effects in β-Cell Survival

There is no doubt that strong survival regulators such as Bcl-2 and Bcl-xL need a tight expression and function regulation, and deficiencies in this control are related to the development of different diseases, namely, different types of cancer [127,128], bone deficiencies [129], asthma complications [130], anemia [131,132,133], and neuronal abnormalities [134,135].

The protective effect of a high expression of Bcl-xL on pancreatic β-cell survival has been demonstrated by us and others. This was firstly reported by Zhou and colleagues, who found that the overexpression of human Bcl-xL in transgenic mouse islets was protective against thapsigargin-induced apoptosis [136]. Subsequent studies demonstrated that Bcl-xL protected β-cells from different insults, as described next. Klein and colleagues demonstrated that the exogenous overexpression of Bcl-xL or its anti-apoptotic BH4 domain protects the NIT-1 mouse β-cell line, as well as human and non-human primate islets, from starvation or staurosporine-induced cell death [137]. Similarly, Bcl-xL-overexpressing RIN-r β-cells were protected against different proinflammatory cytokines [138]. Furthermore, Cunha et al. demonstrated that palmitate-induced lipotoxic death in rat INS-1E β-cells is at least partially due to Bcl-2 and Bcl-xL depletion, and that Bcl-2 knock-down sensitizes INS-1E cells to free fatty acids [121]. Finally, we have recently shown that the effects of Bcl-xL overexpression on β-cell physiology seem to be species-dependent. Bcl-xL overexpression in both INS-1E and human EndoC-βH1 β-cells protects them from both cytokine- and palmitate-induced apoptosis, proving its ability to counteract classical death signals from T1D (i.e., proinflammatory cytokines) and T2D (i.e., palmitate). While Bcl-xL overexpression in rat INS-1E β-cells conferred a 40% protection against proinflammatory cytokines- and palmitate-induced apoptosis, INS-1E cells also showed impaired glucose-induced Ca^2+^ oscillations and insulin secretion. In contrast, Bcl-xL-overexpressing human EndoC-βH1 β-cells conserved their physiological functions while showing an 80% increased protection against proinflammatory cytokines and a complete protection against palmitate-induced apoptosis. Notably, our findings suggest that Bcl-xL protection on human β-cells acts, at least partially, through ER stress alleviation, as Bcl-xL-overexpressing EndoC-βH1 showed a decreased expression of the ER stress markers Bip and XBP1s upon treatment with both insults (i.e., cytokines and palmitate), and reduced Chop expression in response to metabolic stress [19]. Together, these studies present Bcl-xL overexpression as a potential strategy to increase β-cell survival. However, this might not be the case for Bcl-2 overexpression. Allison and colleagues demonstrated that the overexpression of human Bcl-2 was insufficient to prevent or reduce cytotoxic or autoimmune β-cell damage in transgenic mice expressing human Bcl-2 in their islet β-cells. As a result, the onset and incidence of diabetes in three mouse models of T1D—multiple low-dose streptozotocin-induced diabetes, RIP-B7-1 mice, and non-obese diabetic (NOD) mice—were similar between wild-type and transgenic mice overexpressing Bcl-2 [139].

Due to their classical role in ensuring cell survival, many studies have investigated the role of Bcl-2 and Bcl-xL in β-cell endurance against different pro-apoptotic stimuli, such as proinflammatory cytokines [19,140,141,142,143,144], palmitate [19,121,145], and cyclopiazonic acid-induced ER stress [146]. While Bcl-xL-deficient β-cell mice develop even with abnormally high sensitivity to apoptotic insults, including thapsigargin, cytokines, and Fas ligand [141], the lack of this protein seems to be extremely detrimental in human β-cells. A recent study by Loo and colleagues revealed the fundamental role of Bcl-xL in the survival and identity establishment of human pluripotent stem cells during pancreatic specification. Bcl-xL inhibition not only increased the apoptosis rate but also downregulated the expression of pancreatic identity and metabolic genes, preventing the formation of insulin-positive β-like cells and disturbing glycolysis and oxidative phosphorylation [147].

The protection against apoptosis conferred by these proteins can also be detrimental to β-cell survival in the context of diabetes. In NOD mice, Bcl-2 plays an important role in ensuring the survival of activated T lymphocytes against apoptosis induced by IL-2 deprivation. This effect contributes to the autoimmune positive feedback and worsens insulitis and β-cell destruction [148]. Regarding the relationship between the autoimmune attack on β-cells and the expression of Bcl-2 proteins, a recent study from Brozzi and colleagues demonstrated that activated CD4+ T lymphocytes promote apoptosis in targeted β-cells not only by secreting proinflammatory cytokines, but also through the secretion of extracellular vesicles containing Bcl-xL-inhibiting non-coding tRNA fragments [149].

Polymorphisms/mutations in the diabetes-linked paired/homeodomain transcription factor Pax4 have been associated with several forms of diabetes, including T1D, T2D, and monogenic diabetes [150,151]. Pax4 plays a crucial role in β-cell development, proliferation, survival, and function [150,151]. For instance, it promotes β-cell proliferation by regulating the expression of genes involved in cell cycle (e.g., cyclin-dependent kinase inhibitor 2A and c-Myc) [152,153,154]. Moreover, Pax4 enhances β-cell survival by regulating genes involved in ER integrity in response to stress [154] and genes involved in anti-apoptotic pathways, such as Bcl-2 and Bcl-xL [153,155,156]. Studies led by Brun and colleagues reported that *Pax4* upregulated Bcl-xL expression in the rat β-cell line INS-1E in comparison with rat islets [153,156]. This 25-fold higher expression of *Pax4* is not related to cell proliferation. Nevertheless, its higher content in Bcl-xL directly translates to increased cell viability both in basal conditions and in the presence of the proinflammatory cytokines tumor necrosis α (TNFα), interferon gamma (IFNγ), and interleukin-1β (IL-1β). Of note, adenoviral-mediated Pax4 overexpression in human islets induces a small increase in Bcl-xL expression, showing mechanistic differences in the regulation of Bcl-xL expression by Pax4 between humans and rodents [157]. This regulatory effect has also been reported for Bcl-2 in transgenic mice overexpressing Pax4 in β-cells [155]. Interestingly, Hu He and collaborators compared the overexpression of Pax4 with the equivalent overexpression of the diabetes-linked mutant variant of Pax4, Pax4R129W. These researchers found that Pax4 overexpression, but not Pax4R129W, protected animals from streptozotocin-induced hyperglycemia as well as mouse islets from cytokine-induced apoptosis; these protective effects were at least partially due to a three-fold increase in the expression of Bcl-2 [155]. Of note, the overexpression of Pax4 or its mutant version decreased insulin expression, with a long-term effect of insulin content reduction [155]. This is consistent with the suppressing effect of Bcl-2 in glucose tolerance found by Luciani and colleagues [47].

Pro-apoptotic insults, such as proinflammatory cytokines and palmitate, can either directly or indirectly regulate Bcl-2 and Bcl-xL expression in β-cells [52]. Different combinations of proinflammatory cytokines activate distinct pathways that regulate Bcl-2 proteins in β-cells. The combination of IL-1β + IFNγ activates signal transducer and activator of transcription 1 (STAT1), activator protein 1 (AP-1), and NF-κB pathways in β-cells [158,159]. These pathways converge to promote the pro-survival induction of the transcription factor Jun-B and the Bcl-2-related protein A1 [160,161]. Simultaneously, exposure to these cytokines activates the c-Jun N-terminal kinase (JNK) pathway, which upregulates the BH3-only sensitizer DP5 [52]. The pro-survival JunB sequestering of DP5 is surpassed due to Jun-B degradation after JNK activation [161] and the consequential phosphorylation of the E3 ligase Itch [162]. JNK activation also downregulates Mcl-1 [163,164] which, along with Bad activation by IL-1β + IFNγ [165], accelerates DP5-induced β-cell apoptosis. DP5, which is also transcriptionally activated by STAT1 and ER stress [159,166], selectively binds to Bcl-xL, releasing the BH3-only activator Puma, after which this pro-apoptotic Bcl-2 protein binds to Bax and initiates the intrinsic pathway of apoptosis [167]. The combination of TNFα with IFNγ is also regulated by STAT1, which transcriptionally activates Bim, DP5, and Puma, while inactivating Bcl-xL and Mcl-1 [52,164].

Upon treatment with TNFα + IFNγ, Bcl-2 expression remained unaltered, whereas Bcl-xL expression, both at mRNA and protein levels, was diminished in rat INS-1E cells [140]. In contrast, in the mouse NIT-1 cell line, the protein levels of Bcl-2 were enhanced and Bcl-xL expression was not affected by treatment with TNFα alone during 24 h [168]. Mehmeti and colleagues reported that IL-1β alone was sufficient to drastically reduce both the gene and protein expression of Bcl-2 in rat RINm5F cells and rat islets, while Bcl-xL gene expression was upregulated by this proinflammatory cytokine in rat islets. The combination of IL-1β, TNFα, and IFNγ also increased Bcl-xL gene and protein expression in RINm5F cells. Of note, IL-1β-induced Bcl-2 downregulation depended on mitochondrial hydrogen peroxide accumulation [169]. In INS-1 832/13 cells and human islets, treatment with IL-1β, TNFα, and IFNγ—but not glucotoxicity or ER stress—stimulated the expression of microRNA miR-21. This microRNA, in turn, increased β-cell death by degrading *BCL2*/*Bcl2* transcripts and inhibiting Bcl-2 translation [170].

The palmitate-induced activation of the intrinsic apoptotic pathway begins with JNK pathway activation and ATF3 transcription factor induction via the PERK arm of ER stress. PERK activation downregulates Bcl-2, Bcl-xL, and Mcl-1, while simultaneously inducing ATF3 and FoxO3A to upregulate Puma. These events collectively lead to Bax activation and the permeabilization of the mitochondrial outer membrane [52,158]. Although not yet demonstrated in β-cells, it is noteworthy that JNK can directly phosphorylate Bcl-2 and Bcl-xL at their BH3-binding domains [171,172]. As pointed out by Stanley and colleagues [168], this phosphorylation either prevents binding to BH3-only apoptotic proteins or marks Bcl-2 and Bcl-xL for degradation, ultimately releasing BH3-only proteins and increasing the susceptibility to apoptosis. Finally, it has been shown that palmitate blocks the ubiquitin–proteasome system, resulting in ER stress and the downregulation of Bcl-2 and Bcl-xL in MIN6 cells [145].

Bcl-xL is also the object of indirect regulation by other agents. As stated above, the BH3-only sensitizer DP5 binds to and inhibits Bcl-xL in response to IFNγ and IL-1β [159,173] or palmitate [173], favoring apoptosis. On the other hand, exposure to a high concentration of 16.7 mM glucose reduces Bcl-xL expression while leaving Bcl-2 unaffected and increasing the expression of pro-apoptotic members of the family Bad, Bid, and Bik in human islets [174]. This evidence suggests a modulatory effect of high glucose on Bcl-2 proteins towards apoptosis, which could be of special relevance in the T2D context. Bcl-xL downregulation as a vehicular contributor to β-cell damage has also been reported in chemically induced diabetes models. Streptozotocin, a classical inductor of β-cell destruction in murine models of diabetes, exerts its effects by promoting the binding of the BH3-only sensitizer Bad with Bcl-xL while decreasing the interaction with its pro-survival phosphorylated form, P-Bad, thus increasing apoptosis in rat β-cells [175].

An analysis of Bcl-xL expression in α- and β-like cells derived from human induced pluripotent stem cells exposed to IFNα showed that α-like cells present a higher expression of the anti-apoptotic protein Bcl-xL when compared to their β-like counterparts [176]. These findings are consistent with previous results from our group with primary rat α- and β-cells, where we found that the higher basal expression of Bcl-xL in α-cells conferred them resistance to palmitate-induced ER stress compared to β-cells [177]. These findings suggest that the increased expression of Bcl-xL in α-cells both in basal conditions and in response to IFNα could be a crucial feature of α-cell survival in T1D.

Interestingly, transcriptomic data from T1D donor β-cells from the nPOD consortium show a significant increase in Bcl-2 expression in comparison to non-diabetic donors, without changes in Bcl-xL expression [178]. This upregulation might be due to a late but insufficient effort to reestablish a functional β-cell mass during the autoimmune attack, which could be influenced by the already low expression of Bcl-2 in human islets [179]. In fact, previous results from Allagnat and colleagues evidenced that the cytotoxic effectors characteristic of T1D, such as proinflammatory cytokines, affect the expression of Bcl-2 and Bcl-xL as a late event of β-cell apoptosis [164].

The synergistic effect of proinflammatory cytokines towards β-cell death can be aggravated by other regulators of the intrinsic pathway of apoptosis. In brain-dead donors, which are usual candidates for islet transplantation, the increase in expression of the uncoupling protein-2 (UCP2) in islets contributes to accelerating the loss of islet yield and quality by downregulating Bcl-2 both at protein and mRNA levels [180] (Figure 1). Similarly, the cytokine-induced gene *Clic4* sensitizes primary mouse β-cells to apoptosis by reducing the steady state levels of Bcl-2 and Bcl-xL [181] (Figure 1). Well-known non-cytokine regulators of Bcl-2 expression are glucagon-like peptide 1 (GLP-1) agonists such as exedin-4, that has been shown to protect β-cells from ER stress [182]. In fact, exedin-4 has been observed to counteract TNFα-induced apoptosis in INS-1 and MIN6 cells and isolated pancreatic human islets at least partially due to Bcl-2 expression level restoration [183]. Bcl-2 can also be regulated by Bag-1 [184], an anti-cell death protein that binds to and upregulates Bcl-2 activity in hippocampal neurons [185] and cardiomyocytes [186] in the context of diabetes. However, little is known about its effects on pancreatic β-cell survival.

Furthermore, paracrine signals have been proved to regulate Bcl-2 expression. The pancreatic-derived cytokine-like protein PANDER, also known as FAM3B, is secreted by α- and β-cells and induces apoptosis in these cells [187,188,189]. PANDER knockdown has been shown to reduce Bcl-2 expression in colon carcinoma cells, and to decrease cell viability in the β-cell MIN6 cell line, in an arguably similar mechanism [190]. However, these results remain controversial, as a subsequent study by Cao and colleagues showed that PANDER overexpression induces apoptosis without changes in Bcl-2 protein levels in βTC3 cells and mouse islets [191].

In β-cells, cellular stress responses can promote a senescent cell state in the form of replicative senescence activated by a cyclin-dependent kinase inhibitor-induced cell cycle arrest. Although senescent β-cells are a normal feature of adult islets and present enhanced GSIS compared with young pre-senescent β-cells [192,193], they have been found to display DNA double-strand breaks and the activation of DNA damage response in islets from T1D cadaveric donors [194]. The survival of these cells has been reported to act through the upregulation of Bcl-2 expression and to increase during T1D progression in islets from both NOD mice and T1D patients. Interestingly, the pharmacological inhibition of Bcl-2 by the ABT-737 Bcl-2/Bcl-xL inhibitor or the Bcl-2-specific inhibitor ABT-199 eliminated senescent β-cells in NOD mice, preventing diabetes development in this model without affecting the immune cell population or non-senescent β-cells [195]. In this line, a recent study by Rampazzo Morelli and colleagues found that a major senescence-associated secretory phenotype factor of pancreatic β-cells, the growth and differentiation factor 15 (GDF15), is directly linked to the expression of the *BCL2L1* gene in senescent human β-cells. While the antibody neutralization of GDF15 in EndoC-βH5 cells did not affect cell viability, it reduced the expression of the Bcl-xL transcript, among other key features of the senescence-associated secretory phenotype [196].

As described above, we demonstrated that α-cells express higher levels of Bcl-xL than β-cells [177]. In this study, α-cells exhibited a higher mRNA expression of both Bcl-2 and Bcl-xL compared to β-cells, both at baseline and after palmitate exposure. Notably, silencing Bcl-xL significantly increased α-cell sensitivity to palmitate, whereas Bcl-2 knockdown did not enhance susceptibility to palmitate-induced apoptosis [177]. These results suggest that the protective effect in α-cells primarily arises from elevated Bcl-xL expression and may explain the greater resistance of α-cells to metabolic stress in T2D.

Taken together, these findings emphasize the intertwined relationship between Bcl-2 and Bcl-xL protein expression and the pathophysiology of diabetes. In pancreatic endocrine cells, these proteins have a complex role where their expression can be regulated by many different factors, including proinflammatory cytokines and ER stress, but also transcription factors and hormones (e.g., GLP-1).

### 3.2. Bcl-2 and Bcl-xL Effects on β-Cell Function

While Bcl-2 and Bcl-xL overexpression might have beneficial effects on β-cell survival, these anti-apoptotic proteins have also been reported to interfere with insulin secretion and metabolic regulation in murine models. Bcl-xL preserves mitochondrial transcriptional, morphological, and functional integrity under non-apoptotic levels of chronic glucose stress in β-cells [197]. Bcl-2 has been shown to control the fine-tuning of redox balance and ROS signaling in mitochondria [46] and mitochondrial morphology [121]. While these beneficial effects on mitochondrial homeostasis would identify a good starting point for hormone secretion regulation, mice overexpressing high levels of Bcl-xL (i.e., >10-fold increase) presented impaired intracellular Ca^2+^ signaling and mitochondrial ATP production. These changes led to reduced glucose-stimulated insulin secretion (GSIS) [136].

Later, it was reported that the adenoviral-induced overexpression of Pax4 increased the expression of Bcl-xL in rat islets. This effect was also concomitant to decreased mitochondrial Ca^2+^ concentration and therefore ATP production and GSIS [153]. Curiously, the overexpression of Pax4 did not alter insulin or glucagon expression in the rat islets. Conversely, transgenic mice overexpressing Pax4 exhibited a significative increase in Bcl-2 mRNA but not in Bcl-xL, and they were protected from streptozotocin-induced hyperglycemia [155].

Nevertheless, most evidence points towards an inhibitory activity of Bcl-2 and Bcl-xL in β-cell metabolism. In a very thorough study, Luciani and colleagues used knock-out mouse models of both Bcl-2 and Bcl-xL to demonstrate that the lack of these proteins significantly increased glucose-stimulated intracellular Ca^2+^ and metabolic responses, concomitant to increased glucose-induced insulin secretion. Furthermore, these effects could also be obtained by the small-molecule antagonism of Bcl-2 and Bcl-xL, and through the conditional ablation of Bcl-xL on adult β-cells [47,198] (Figure 1). We have recently confirmed this effect on Bcl-xL-overexpressing rat INS-1E β-cells, which showed dampened intracellular Ca^2+^ oscillations and insulin secretion in response to glucose and forskolin. However, the overexpression of Bcl-xL did not affect the Ca^2+^ response to glucose and GSIS in human EndoC-βH1 β-cells, suggesting species-specific mechanisms in the regulation of cell function by Bcl-2 proteins [19]. The observed lack of physiological effects of Bcl-xL overexpression in human β-cells might also suggest an existing threshold of Bcl-xL overexpression for Ca^2+^ homeostasis alterations. As reported by Zhou and collaborators, an over 10-fold overexpression of Bcl-xL might be enough to induce such Ca^2+^ homeostasis impairment, but a 2- to 3-fold increase has no effects on glucose tolerance [136]. Similarly, our results show that a 7-fold increase in Bcl-xL expression affects intracellular Ca^2+^ and insulin secretory response to glucose in rat INS-1E cells. In contrast, a 2.5- to 5-fold induction of Bcl-xL is enough to significantly protect human β-cells from diabetogenic insults without reaching the physiologically disturbing threshold. This highlights the importance of fine-tuning the dose–response of Bcl-xL overexpression for future therapeutic approaches.

Discrepancies in outcomes could be due to intrinsic differences between animal models. For example, human Pax4 DNA binding activity has been found to be lower than that of the mouse homologue in human islets, as can be seen in the fact that the overexpression of mouse Pax4 induces human islet cell proliferation, while the human homologue lacks this effect [157]. Additionally, it is difficult to compare effects between models when Bcl-2/Bcl-xL overexpression levels have great disparity: Zhou and colleagues reached a 10-fold increase in Bcl-xL expression [136], much higher than the increased expression of Bcl-xL by 2.7-fold in rat islets overexpressing Pax4 found by Brun and colleagues [153] and the 3-fold increase in Bcl-2 obtained in transgenic mice by Hu He and coworkers [155]. Conversely, Allison and colleagues observed that human Bcl-2 overexpression in mice had no effects on β-cell function or resistance to β-cell apoptosis [139]; of note, the level of overexpression was not clearly established in this study. The results obtained by our group corresponded to a 7-fold increase in Bcl-xL expression in rat INS-1E cells and a 5-fold increase in human EndoC-βH1 cells [19]. Our results in the rat cell line concur with those obtained by Brun and colleagues, but this is a field in which more research is needed, as it is possible that a certain level of Bcl-2/Bcl-xL hyperexpression is needed to interfere with Ca^2+^ and mitochondrial homeostasis, and this threshold might be different for each animal or human model.

## 4. Therapeutic Opportunities

Due to their role in β-cell survival, a growing body of evidence suggests that Bcl-2 and Bcl-xL represent exciting therapeutic candidates to enhance β-cell robustness in the context of T1D and T2D. As discussed by Singh and colleagues, enhancing β-cell robustness may protect individuals predisposed to T1D or prolong the honeymoon phase in recent-onset T1D patients. In T2D patients, it could help prevent insulin dependency, thereby reducing complications and improving long-term outcomes [199].

Based on the literature, we discuss two possible therapeutic approaches that might be used for future clinical studies.

### 4.1. Gene Therapy

Adeno-associated virus (AAV) vectors have emerged as the preferred choice in clinical trials due to their broad tissue tropism and safety. In addition, their non-pathogenic nature, minimal genome integration, and long-term transgene expression contribute to their effectiveness in enhancing cellular entry and transduction [200,201,202]. Therefore, the use of an AAV-based gene delivery system to increase Bcl-xL expression in β-cells could be considered. More specifically, we could use a pancreas-selective adeno-associated virus (serotype 8) encoding for *BCL2L1* under the control of the insulin promoter to enhance Bcl-xL expression in β-cells, as previously shown for other proteins [199,203].

Despite the positive safety profile, patients receiving AAV-based gene therapy would need continuous monitoring. This should focus on the potential long-term risks related to the expression of an anti-apoptotic protein, which includes the risk of tumor development [201]. Current AAV-based gene therapies undergoing clinical trials demonstrate favorable safety profiles, with no significant evidence of tumorigenesis. Additionally, the predominantly epichromosomal nature of AAV-delivered DNA [204] reduces the likelihood of genomic integration, further mitigating oncogenic risks. It is worth mentioning that studies using transgenic mice overexpressing Bcl-xL in islets/β-cells have not reported tumor growth, suggesting that these models remained cancer-free during experiments [136,205].

Beyond in vivo gene therapy, an alternative use for Bcl-xL could be to prolong graft survival in pancreatic islets for transplantation. For this, either AAV-based gene therapy [206] or CRISPR/dCas9-based enhancer activation [207] could be employed to induce Bcl-xL overexpression in human islets, which could subsequently be transplanted into individuals with T1D.

### 4.2. Bcl-2/Bcl-xL Inhibitors

The inhibition of specific Bcl-2 proteins has been described as a promising therapeutic strategy for cancer. In recent years, researchers have combined nuclear magnetic resonance (NMR)-based screening, fragment chemistry, and structure-based drug design to generate a class of compounds known as BH3 mimetics [15,16]. BH3 mimetics are small molecules that mimic the binding of the BH3-only initiator proteins to the pro-survival members, effectively displacing native BH3-only proteins. Interestingly, it has been reported that these inhibitors can induce apoptosis with some specificity in cells exhibiting high levels of Bcl-2 or related members such as Bcl-xL and Bcl-w [51].

Most studies in β-cells centered on the use of ABT-737 (a Bcl-2, Bcl-xL, and Bcl-w inhibitor), ABT-263 (a Bcl-2 and Bcl-xL dual antagonist), and ABT-199 (a highly selective Bcl-2 inhibitor) (Table 1). Initial studies showed that ABT-737 induced apoptosis in human islet cells, primary rat β-cells, and INS-1E cells. Moreover, Puma silencing partially reduced ABT-737-induced apoptosis, indicating that Bcl-2/Bcl-xL inhibition triggers Puma-mediated β-cell death [142]. Several years later, it was reported that the specific inhibition of Bcl-2 by ABT-199 did not induce β-cell death after treatment for up to 24 h. This suggests that, compared to other anti-apoptotic members of the Bcl-2 family, Bcl-2 plays a minimal role in preventing apoptosis in β-cells under oxidative stress [46].

The therapeutic potential of Bcl-2/Bcl-xL inhibitors in diabetes emerged in 2019, when two complementary studies introduced the use of BH3 mimetics as senolytic compounds, which are drugs that selectively induce apoptosis in senescent cells [195,208]. First, Thompson and colleagues demonstrated that ABT-737 and ABT-199 mainly targeted and eliminated senescent β-cells in mouse islets without altering the abundance of the immune cell types (e.g., macrophages, effector T cells, and B cells). In addition, compared to control animals, treatment with ABT-199 in normoglycemic NOD mice suppressed diabetes development [195]. A couple of months later, a study by Aguayo-Mazzucato et al. demonstrated that oral treatment with ABT-263 alleviated hyperglycemia and enhanced the β-cell gene expression profile in animals treated with an insulin receptor antagonist (a drug-induced insulin-resistant mouse model). In human islets, the senescent cell subpopulation showed increased cell death after ABT-263 treatment [208]. Altogether, these results suggest that in T1D and T2D, β-cells become senescent, develop a senescence-associated secretory profile, and resist apoptosis through the upregulation of Bcl-2. Consequently, targeting the elimination of senescent β-cells with BH3 mimetics arrests the immune- and metabolic-mediated loss of functional β-cells and inhibits the progression of T1D and T2D by preserving β-cell mass.

Regarding their use in humans, Aguayo-Mazzucato and colleagues recognize that the primary limitation of senolytic therapies is their broad, non-specific targeting of cells and tissues, as they act on pathways that can be upregulated across various cell types. Additionally, senolytic drugs with greater potency and oral efficacy with manageable side effects must be developed. Finally, continuous understanding of senescence biology will aid in the development of effective therapeutics, paving the way for optimal and personalized interventions [208,209].

## 5. Limitations/Gaps in Knowledge and Future Perspectives

Although significant progress has been made in recent years, several questions remain unanswered concerning the roles of Bcl-2 and Bcl-xL in β-cell survival and physiology. Here, we highlight two of the major gaps in our current knowledge and areas where further research is needed to bridge these gaps.

### 5.1. Limited Research on Human Models

It is well known that rodent and human β-cells differ in several aspects, including susceptibility to cytotoxic agents, electrophysiological properties, and the regulation of insulin secretion [210,211]. Despite these differences, the evidence discussed herein suggests that Bcl-2 and Bcl-xL play a conserved role in β-cell survival across species. However, some differences can be seen regarding protection against some stressors (e.g., cytokines and palmitate). For instance, while Bcl-xL overexpression fully prevented palmitate-induced apoptosis in human EndoC-βH1 β-cells, rodent β-cells (INS-1E and mouse islets) were only partially protected by Bcl-2/Bcl-xL overexpression [19,145]. This suggests that palmitate-induced apoptosis may have a component that does not depend on the role of Bcl-2 proteins in rodents. Despite the absence of data from human islets, the gold standard in β-cell research, it is reasonable to state that Bcl-xL plays a protective role in both rodent and human β-cells against different pro-apoptotic stimuli.

Regarding β-cell function, the literature lacks an in-depth comparative analysis of how Bcl-2 and Bcl-xL influence β-cell electrophysiology, exocytosis dynamics, mitochondrial metabolism, and insulin secretion in rodents and humans. To our knowledge, only two studies have investigated the physiological effects of Bcl-2 and Bcl-xL in human β-cells and both only analyzed intracellular Ca^2+^ signaling [19,47]. Luciani et al. showed that Bcl-2/Bcl-xL inhibition rapidly induced significant Ca^2+^ fluctuations in mouse and human islet cells [47]. We, on the other hand, showed that Bcl-xL overexpression in rat INS-1E cells slightly reduced intracellular Ca^2+^ responses and glucose-stimulated insulin secretion, whereas these effects were not observed in the human EndoC-βH1 cells [19]. As discussed above, it remains unclear whether this lack of effect in human cells is due to the level of Bcl-xL expression or to differences between humans and rodents.

To overcome one of the main limitations of human studies, namely, the acquisition of human islets, we believe that future research should focus on alternative approaches, such as organoids and β-cells derived from human pluripotent stem cells [212], as well as β-cell lines that closely resemble primary human cells (e.g., EndoC-βH5 cells) [213].

### 5.2. Limited Preclinical Studies in Mouse Models of T1D and T2D

In this review we described several studies in which Bcl-2 or Bcl-xL overexpression in islets/β-cells conferred β-cell protection against pro-apoptotic stressors. Nonetheless, none of these studies investigated whether Bcl-2/Bcl-xL overexpression was effective in preventing diabetes development in animal models for T1D (e.g., NOD or RIP-B7.1 mice) or T2D (e.g., high-fat diet-induced diabetes or db/db mice). These preclinical studies are crucial to provide evidence of the feasibility of using Bcl-2/Bcl-xL overexpression as a therapeutic approach (as discussed in Section 4). Additionally, we would have the opportunity to further investigate the threshold of Bcl-2/Bcl-xL expression that protects against a diabetogenic environment without disturbing β-cell physiology. Lastly, these studies will be essential to evaluate whether targeting these proteins therapeutically will eventually trigger off-target effects.

As discussed above, excessive Bcl-xL expression (>10-fold) disrupts intracellular Ca^2+^ homeostasis, impairing insulin secretion in rodent β-cells [136]. Furthermore, excessive Bcl-2/Bcl-xL levels may hinder the natural apoptosis process, allowing dysfunctional β-cells to persist, ultimately impairing glucose-stimulated insulin secretion. Finally, by interacting with Beclin-1, Bcl-2 inhibits autophagy, a process essential for β-cell survival and function [214]. Hence, prolonged Bcl-2 overexpression may lead to autophagy imbalance, worsening β-cell health and promoting apoptosis. To mitigate these potential risks, we suggest the use of molecular tools that allow the fine-tuning of Bcl-2/Bcl-xL expression, such as inducible expression systems (e.g., Tet-on/Tet-off) and CRISPR-based approaches. Ideally, Bcl-2/Bcl-xL levels should match those observed in human α-cells, which are approximately 3–4 times higher than basal levels (HPAP database: https://hpap.pmacs.upenn.edu/).

## 6. Conclusions

In the present review, we summarize the different roles of Bcl-xL and Bcl-2 in survival and function, emphasizing their roles in β-cells, where they serve a dual impact on physiological processes, such as Ca^2+^ homeostasis and insulin secretion, as well as cell survival. The lack of studies on human models represents a significant gap, as human β-cells respond differently to stress compared to rodent cells. Addressing this gap is essential for translating findings into therapeutic approaches targeting human β-cell preservation under proinflammatory or metabolic conditions. Recent evidence, however, shows the ability of Bcl-2 and Bcl-xL to protect human β-cells without impairing insulin release, introducing a promising avenue for β-cell-specific therapies aimed at preventing β-cell loss during diabetes progression. Altogether, these findings suggest that targeting Bcl-2 and Bcl-xL could represent a viable strategy to mitigate β-cell dysfunction and death, contributing to advancements in diabetes treatment.

## Figures and Tables

**Table 1:** Preclinical studies using Bcl-2/Bcl-xL inhibitors in the context of β-cells/diabetes.

Inhibitors	Models	References
ABT-737	Human islets, primary rat β-cells, and rat INS-1E cells	[142]
ABT-199	Mouse islets and mouse MIN6 cells	[46]
ABT-199, ABT-737	Mouse islets	[195]
ABT-263	Mouse and human β-cells	[208]

ABT-199 is also known as Venetoclax, and ABT-263 is known as Navitoclax.
